# Retraction Note: The specific MYB binding sites bound by *Ta*MYB in the *GAPCp2/3* promoters are involved in the drought stress response in wheat

**DOI:** 10.1186/s12870-020-02782-w

**Published:** 2020-12-21

**Authors:** Lin Zhang, Zhiqiang Song, Fangfang Li, Xixi Li, Haikun Ji, Shushen Yang

**Affiliations:** College of Life Sciences, Northwest A&F University, Yangling, 712100 Shaanxi China

**Retraction note to: BMC Plant Biol 19, 366 (2019)**

**https://doi.org/10.1186/s12870-019-1948-y**

The editor of BMC Plant Biology has retracted this article [1] due to concerns about the figures. Specifically, it was brought to the attention of the journal that:

- The TaGAPCp3/TaMyb panel of Fig. 6B is similar to TaGAPCp2P-1/TaMyb panel of Fig. 9B in the same article and Fig. 5D of a different publication by the some of the same authors [2];
- TaGAPCp3P-3/TaMyb of Fig. 9B appears to overlap with the TaGAPC1P-3/TaWRKY40 panel in Fig. 6C of another paper by some of the same authors [3];
- Fig. 5A (OE-2 and OE-3; drought 25d) appears to overlap with Fig. 2A (OE; drought 25d) of another article [3]

Due to the problematic figures, the editor considers the data presented to be unreliable.

All authors agree to this retraction and apologize to the readers for any inconvenience caused.
